# Analysis of the improved mechanism of *Rhodobacter sphaeroides* VK-2-3 coenzyme Q10 by reverse metabolic engineering

**DOI:** 10.3389/fmicb.2024.1410505

**Published:** 2024-07-04

**Authors:** Long Zhang, Le-yi Wang, Yi-jun Han, Yan-xin Liu, Yong-li Li, Jian-hua Hu, Zhi-jie Tian, Zhan-ying Liu

**Affiliations:** ^1^Inner Mongolia Energy Conservation and Emission Reduction Engineering Technology Research Center for Fermentation Industry, Hohhot, China; ^2^Engineering Research Center of Inner Mongolia for Green Manufacturing in Biofermentation Industry, Hohhot, China; ^3^College of Chemical Engineering, Inner Mongolia University of Technology, Hohhot, China; ^4^Shenzhou Biotechnology Co., Ltd., Hohhot, China

**Keywords:** coenzyme Q10, ^12^C^6+^ heavy ion beam, high-voltage prick electric field, nicotinamide adenine dinucleotide-dependent dehydrogenase, *Rhodobacter sphaeroides*

## Abstract

Coenzyme Q10 (CoQ10) is an essential medicinal ingredient. In this study, we obtained a high-yielding mutant strain of CoQ10, VK-2-3, by subjecting *R. sphaeroides* V-0 (V-0) to a ^12^C^6+^ heavy ion beam and high-voltage prick electric field treatment. To investigate the mutation mechanism, the complete genomes of VK-2-3 and V-0 were sequenced. Collinearity analysis revealed that the nicotinamide adenine dinucleotide-dependent dehydrogenase (NAD) gene underwent rearrangement in the VK-2-3 genome. The NAD gene was overexpressed and silenced in V-0, and this construct was named RS.NAD and RS.ΔNAD. The results showed that the titers of CoQ10 in the RS.NAD and RS.ΔNAD increased and decreased by 16.00 and 33.92%, respectively, compared to those in V-0, and these differences were significant. Our results revealed the mechanism by which the VK-2-3 CoQ10 yield increases through reverse metabolic engineering, providing insights for genetic breeding and mechanistic analysis.

## 1 Introduction

Coenzyme Q10 (CoQ10), also known as ubiquinone, is a transferring hydrogen carrier in the redox respiratory chain and a stabilizer of cell membranes (Lenaz and Genova, 2009). It is widely used in the pharmaceutical, food, and cosmetic additive industries (Campagnolo et al., 2017; Hernández-Camacho et al., 2018). Currently, microbial fermentation is the primary method for producing CoQ10. *Rhodobacter sphaeroides* (*R. sphaeroides*) is the primary producer of CoQ10 (Yen et al., 2010). Furthermore, some traditional mutagenesis methods can also increase the CoQ10 titer (Yao et al., 2021).

With the repeated use of traditional mutagenesis, microorganisms have developed tolerance, leading to a stable mutagenic effect. The ^12^C^6+^ heavy-ion beam and high-voltage prick electric field mutagenesis are two new methods of mutagenesis. The combination of these two methods has not been reported in the mutation breeding of *R. sphaeroides*. Compared to other traditional mutagenesis methods, they offer advantages such as a high mutation rate, a high relative biological effect, high linear energy transfers, and being less prone to revert mutations (Chen et al., 2017). Moreover, both methods had a positive mutagenic effect on other microorganisms. For example, a mutant strain of *Saccharomyces cerevisiae* with 73% greater β-glucan production was obtained through ^12^C^6+^ heavy-ion beam treatment (Li et al., 2017). *Soybean* seeds were treated with a high-voltage prick electric field, and the results indicated that the seed performance of the 2 kV electric field treatment group was significantly better than that of the control group (Liu and Sun, 2008).

Although mutations can create beneficial strains, their use and promotion without analyzing the underlying mechanism will be limited. Reverse metabolic engineering can effectively address this issue. This approach involves using reverse thinking to analyze cells. First, the desired phenotype is achieved. The relevant software was used to analyze the genes that affect the phenotype. Finally, genetic engineering techniques can be used to create an organism with desired traits. This approach forms the basis for expanding the application and scope of the benefit (Bailey et al., 1996; Cacciottolo et al., 2010; Zhou et al., 2015). In this study, we employed two new mutagenesis techniques, a ^12^C^6+^ heavy-ion beam and a high-voltage prick electric field, to induce mutations in *R. sphaeroides* V-0 (V-0). Reverse metabolic engineering was used to analyze the CoQ10 production mechanism of VK-2-3. A crucial gene, nicotinamide adenine dinucleotide-dependent dehydrogenase (NAD), was finally discovered. The gene was then overexpressed and knocked down to study the mechanism of VK-2-3 CoQ10 enhancement. Our study will serve as a reference for mutation breeding and analysis of the mechanisms underlying the increased yield of target products.

## 2 Materials and methods

### 2.1 Strains and culture media

V-0 and VK-2-3 (CCTCC M 2021735) were preserved at this laboratory. Media A was used to activate *R. sphaeroides*, which contained 3 g/L glucose, 8 g/L yeast extract powder, 2 g/L NaCl, 1.3 g/L KH_2_PO_4_, 0.25 g/L MgSO_4_·7H_2_O, 20 g/L agar powder, and 1 mL/L auxiliary liquid (1 g/L nicotinic acid, 1 g/L aneurine hydrochloride, and 0.015 g/L biotin). The pH was adjusted to 7.16 with 10% NaOH. The seed liquid of *R. sphaeroides* was cultivated in media B, which contained 3 g/L glucose, 8 g/L yeast extract powder, 2 g/L NaCl, 1.3 g/L KH_2_PO_4_, 0.25 g/L MgSO_4_·7H_2_O, 0.01 g/L CoCl_2_·6H_2_O, and 1 mL/L auxiliary liquid, and the pH was adjusted to 7.0 with 10% NaOH. The fermentation broth of *R. sphaeroides* was cultivated in media C, which contained 35 g/L glucose, 12 g/L corn syrup powder, 3 g/L C_5_H_8_NO_4_Na, 3 g/L NaCl, 3 g/L (NH_4_)_2_SO_4,_ 3 g/L KH_2_PO_4_, 12.5 g/L MgSO_4_·7H_2_O, 12 g/L CaCO_3_, 0.01 g/L CoCl_2_·6H_2_O, and 1 mL/L auxiliary liquid, and the pH was adjusted to 7.0 with 10% NaOH. *R. sphaeroides* was cultured at 32°C and 220 rpm. The pBBR1MCS-4 plasmid in *Escherichia coli* (*E. coli*) DH5α was provided by Prof. Feng Li from Tianjin University. The bacteria were cultured at 37°C in Luria–Bertani media (LB, 10 g/L tryptone, 5 g/L yeast extract, and 10 g/L NaCl) supplemented with 50 mg/mL ampicillin at a ratio of 1:1000 for plasmid extraction. The pK18mobSacB plasmid in *E. coli* DH5α, which was purchased from Shanghai Zeye Biotechnology Co., Ltd., was used for gene knockout. It is resistant to kanamycin, with a mother liquor concentration of 50 mg/mL, and the inoculation ratio was the same as that of ampicillin. *E. coli* DH5α and S17-1 competent cells were obtained from Shanghai Bohu Biotechnology Co., Ltd.

### 2.2 Screening for the minimum inhibitory concentration of resistant substances

Resistant plates with varying concentrations of p-hydroxybenzoic acid (P), vitamin K3 (V), sodium azide (N), chloramphenicol (L), roxithromycin (R), and kanamycin (K) were prepared, and the cultured seed solution was evenly coated onto the plates. The culture was incubated for 5–6 days at 32°C with 35 ~ 45% humidity while avoiding light. The strains were observed to determine their minimum inhibitory concentration.

### 2.3 ^12^C^6+^ heavy ion beam primary and secondary irradiation

The National Laboratory of Heavy Ions at the Lanzhou Institute of Modern Physics operates a heavy ion accelerator with a bombardment frequency of 1,000 ions/s. The seed culture in the logarithmic growth stage was placed in an irradiation dish and exposed to a ^12^C^6+^ ion beam at a rate of 40 Gy/min. The irradiation doses used were 25 Gy, 50 Gy, 75 Gy, 100 Gy, 125 Gy, 150 Gy, 175 Gy, 200 Gy, 225 Gy, 250 Gy, 275 Gy, and 300 Gy. The numbers 1 ~ 12 represent the 12 radiation doses. For example, V-4 indicates that the bacterial solution was exposed to a 100 Gy radiation dose and had V resistance. After ^12^C^6+^ heavy-ion beam primary mutagenesis, the gene with the highest CoQ10 titer was selected for secondary mutagenesis. The bacterial liquid treatment method and irradiation rate were consistent with those used for primary mutagenesis. The irradiation doses were 100 Gy and 225 Gy. The VL-4 strain exhibited dual resistance and was irradiated at a dose of 100 Gy.

### 2.4 High-voltage prick electric field mutagenesis

The cover of the plate medium with a single colony was removed, and the colonies were placed in a high-voltage prick electric field. The system consists of a multineedle plate electrode and a high-voltage power supply. The upper electrode consisted of a multineedle array with 2 cm needles spaced 4 cm apart, while the lower electrode was an aluminum plate. The distance between the electrodes was 6 cm. The voltage range can be adjusted from 0 ~ 70 kV. The mutagenesis voltages were 10 kV, 13 kV, 16 kV, and 19 kV. The mutagenesis times were 10 and 20 min for a total of 8 groups. The 1–1, 1–2, 1–3, and 1–4 corresponded to 10 min treatments in electric fields of 10 kV, 13 kV, 16 kV, and 19 kV, respectively. 2–1, 2–2, 2–3, and 2–4 denote 20 min of treatment in electric fields of 10 kV, 13 kV, 16 kV, and 19 kV, respectively. Two letters indicate that the strain had dual resistance. In addition, V (5 mg/L) and K (40 mg/L) were added to the V-0 strain not subjected to compound mutagenesis, which was named RS.VK.

### 2.5 Screening method for high-yielding CoQ10 mutants

The bacterial suspension from the ^12^C^6+^ heavy ion beam and high-voltage prick electric field mutagenesis was diluted to a reasonable concentration and coated with plate media A containing the resistant substance. After the colonies had grown, round and green single clones (excluding red mutants) were picked from the resistant plate and inoculated into media B for cultivation. A total of 6 mL of seed solution cultured to the logarithmic stage was added to 500 mL conical flasks containing 60 mL of media C. The flasks were incubated at 32°C and 220 rpm for 72 h in the dark. Rescreening was conducted using high-performance liquid chromatography (HPLC), and the one with the greatest increase in the CoQ10 titer was chosen for further research.

### 2.6 Methods for observing cell morphology

The cell morphology of V-0 and VK-2-3 was observed using scanning electron microscopy (SEM, JSM-IT800, JEOL Co., Ltd., Beijing). The V-0 and VK-2-3 bacterial solutions were taken out of the ultra-low-temperature refrigerator, thawed gradually, and then inoculated into 20 mL of media B with a 1% inoculum, respectively, and 32°C, 220 rpm shaking culture to logarithmic stage. Taking 5 mL of bacterial solution in the logarithmic growth phase, centrifuging it at 3000 rpm for 3 min, and discarding the supernatant. After being washed three times with phosphate buffer solution, the cells were fixed with a 2.5% glutaraldehyde solution and refrigerated at 4°C for 4 h. Subsequently, the cells were washed again with a phosphate buffer solution. The centrifugation conditions for each wash were 8,000 rpm for 5 min, as were the subsequent washes. The bacteria were dehydrated using anhydrous ethanol at concentrations of 30, 50, 70, 80, 90, and 100% for 15–20 min. After washing once with a mixed solution of ethanol and tert-butanol (V: V = 1:1), the bacteria were washed twice with tert-butanol for 20 min each and refrigerated at 4°C for 30 min. After freeze-drying and gold-spraying the samples, their cell morphology was observed using scanning electron microscopy.

### 2.7 Comparative genomic analysis of V-0 and VK-2-3

Samples of V-0 (NCBI Bioproject: PRJNA818168) and VK-2-3 (NCBI Bioproject: PRJNA818446) were sent to Novogene Co., Ltd., for genome sequencing. Mumer software was used to annotate the scaffold sequences in the sequencing results, and GenBank format files for V-0 and VK-2-3 were obtained. The scaffold sequence was then imported into mauve software for collinearity analysis. After combining the GenBank file annotation information for V-0 and VK-2-3 with the results of collinearity analysis, we identified the differential gene. The genome of the standard strain *R. sphaeroides* 2.4.1 was used as a reference.

### 2.8 Construction and transformation of plasmids for overexpression and knockout

The bacterial genome extraction kit was purchased from Real-Times (Beijing) Biotechnology Co., Ltd., China. The Plasmid Miniprep Purification Kit and T4 DNA ligase were purchased from Thermo Fisher Scientific, United States. PCR reagents, agarose gel DNA purification kits, and restriction endonucleases (HindIII and BamHI) were obtained from Takara, China. The detailed experimental procedures are described in the Supplementary material, the sequences of primers used in this study are shown in Supplementary Table S1, and the results of the recombinant vector construction are shown in Supplementary Figures S1, S2.

### 2.9 Determination of fermentation parameters

The CoQ10 titer, yield, production performance, fermentation broth residual sugar concentration, cell dry weight, and relative expression of the NAD gene were determined based on our previous studies (Zhang et al., 2022).

### 2.10 Methods of data analysis

All the above experiments were conducted in three biological replicates, and the results are presented as the means ± error bars. GraphPad Prism was used for plotting, and SAS 9.2 was used to test for statistical significance. Relative expression levels were calculated using the 2^−∆∆Ct^ method.

## 3 Results and discussion

### 3.1 Study on the minimum inhibitory concentration of different substances against *Rhodobacter sphaeroides*

**Figure 1:**
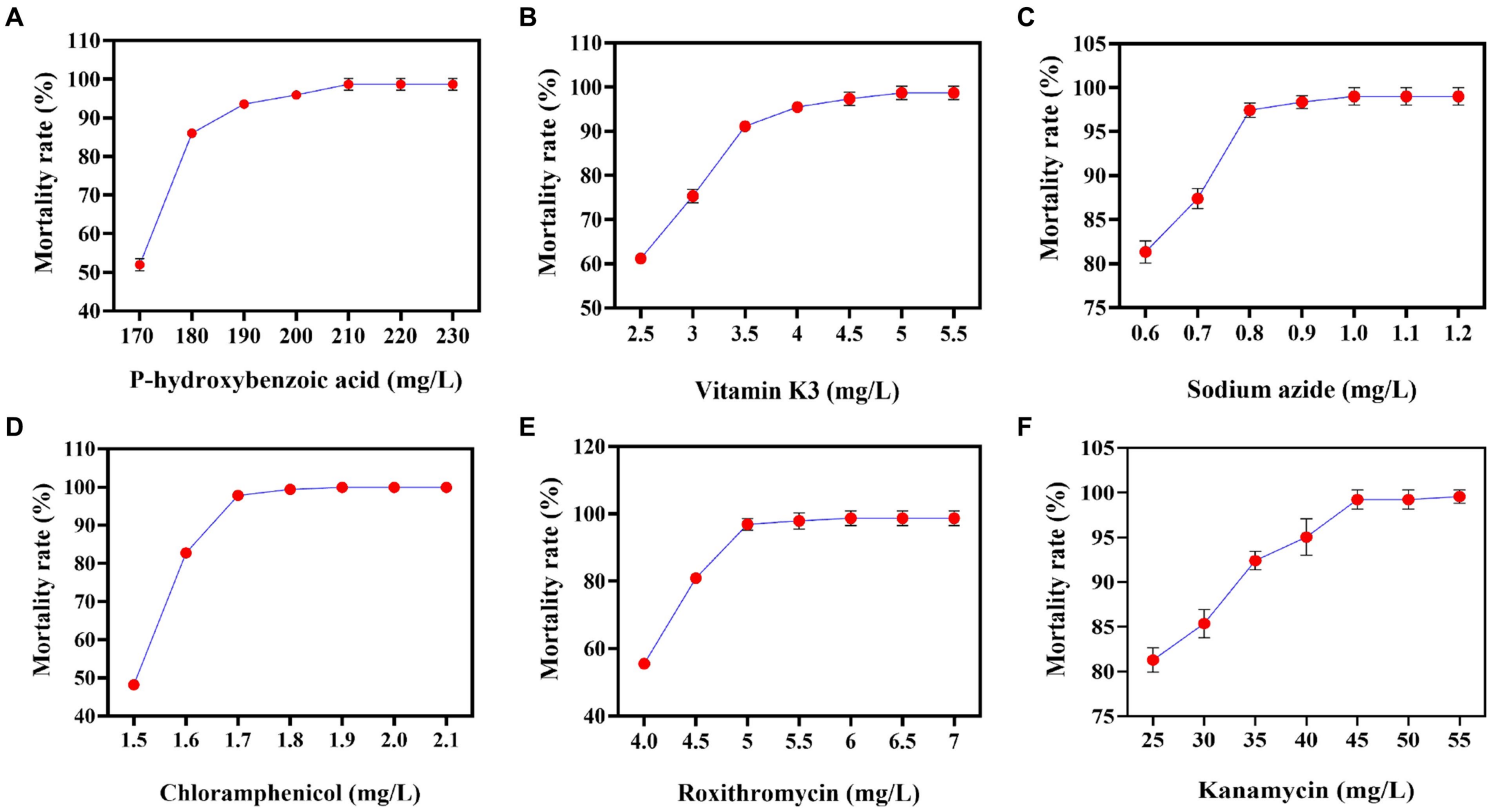
Correlations between the different concentrations of resistant substances and mortality rates. **(A)** P-hydroxybenzoic acid mortality rate curve. **(B)** Vitamin K3 mortality rate curve. **(C)** Sodium azide mortality rate curve. **(D)** Chloramphenicol mortality rate curve. **(E)** Roxithromycin mortality rate curve. **(F)** Kanamycin mortality rate curve.

CoQ10 is a lipid-soluble antioxidant that enhances cellular nutrition in human cells. It has been found to improve human immunity, boost antioxidant activity, and slow aging (Zhou et al., 2023). It is widely used in medicine to treat cardiovascular diseases (Sumbalová et al., 2023). It cannot be quickly and intuitively screened due to its intracellular nature and lack of a color reaction or transparent circle to indicate its yield. As a result, in order to screen for strains with high CoQ10 titers, some researchers have first conducted resistance screening to alleviate substrate feedback inhibition or enhance antioxidant capacity. Subsequently, fermentation experiments were carried out using the resistant strains, and the titer of CoQ10 was quantified using HPLC (Shi et al., 2006). In this study, P-, V-, N-, L-, R-, and K-resistant plates were used to screen for minimum inhibitory concentrations of *R. sphaeroides*. They can alleviate self-precursor substance inhibition, relieve product feedback inhibition, increase cytotoxicity tolerance, decrease the electron transfer rate, scavenge self-free radicals, and enhance cellular antioxidant capacity. The results indicated that the minimum inhibitory concentrations of P, V, N, L, R, and K for V-0 were 210 mg/L, 5 mg/L, 1.0 mg/L, 1.8 mg/L, 6 mg/L, and 40 mg/L, respectively (Figure 1). Although the previous results differed from those of this study, this may be due to variations in the minimum inhibitory concentration based on media composition and strain source (Ke et al., 2015).

### 3.2 ^12^C^6+^ heavy-ion and high-voltage prick electric field mutagenesis combined with resistance screening for CoQ10 high-yielding mutant strains

**Figure 2:**
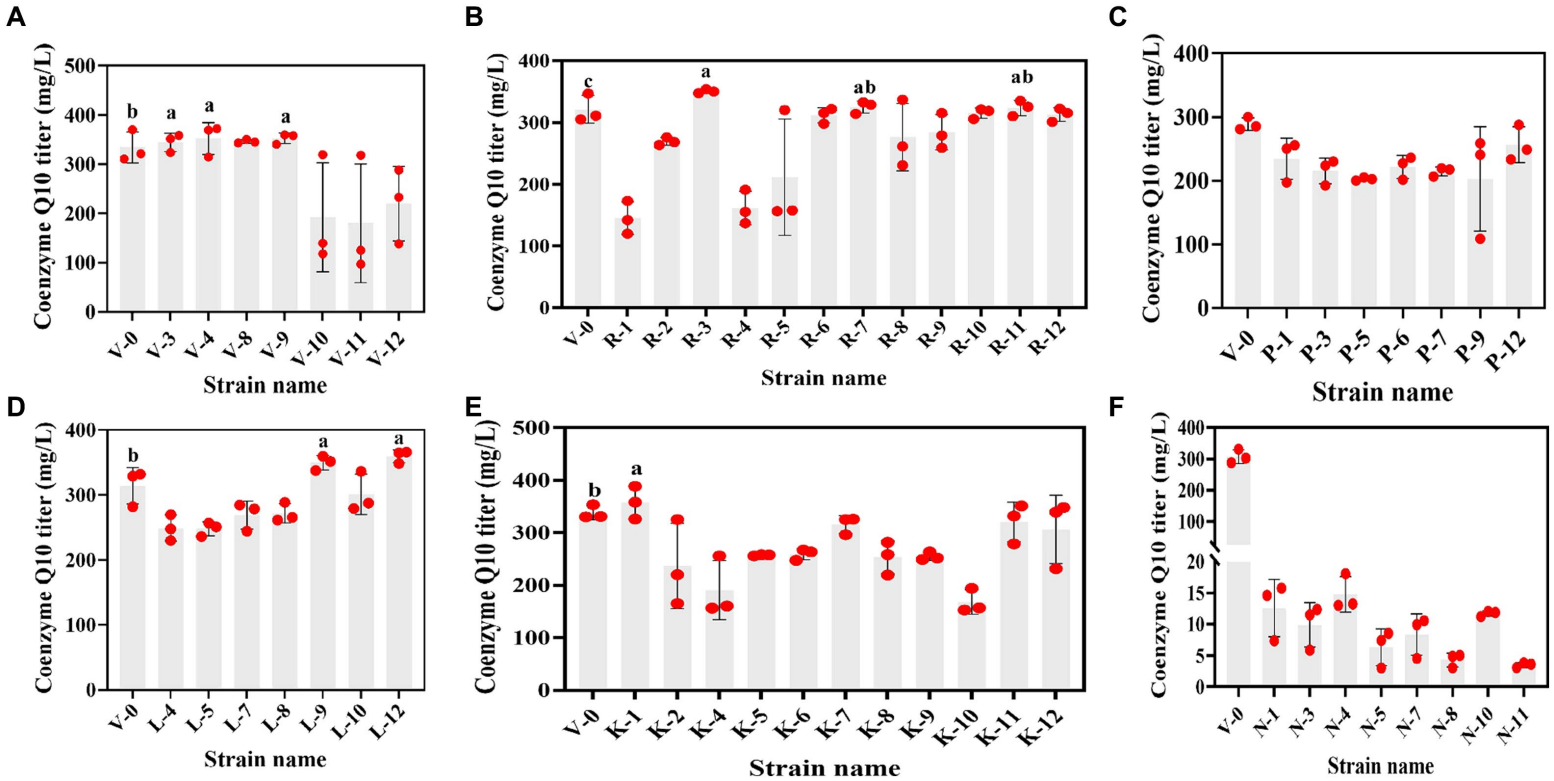
The relationship between resistant mutant strains and the CoQ10 titer. **(A–F)** are vitamin K3, roxithromycin, p-hydroxybenzoic acid, chloramphenicol, kanamycin, and sodium azide resistant mutant strains that were obtained under ^12^C^6+^ heavy ion primary mutagenesis conditions, respectively. The different lower-case letters in the shoulder note indicate that there was a significant difference between the two comparison combinations (*p* < 0.05, Student’s *t*-test).

**Figure 3:**
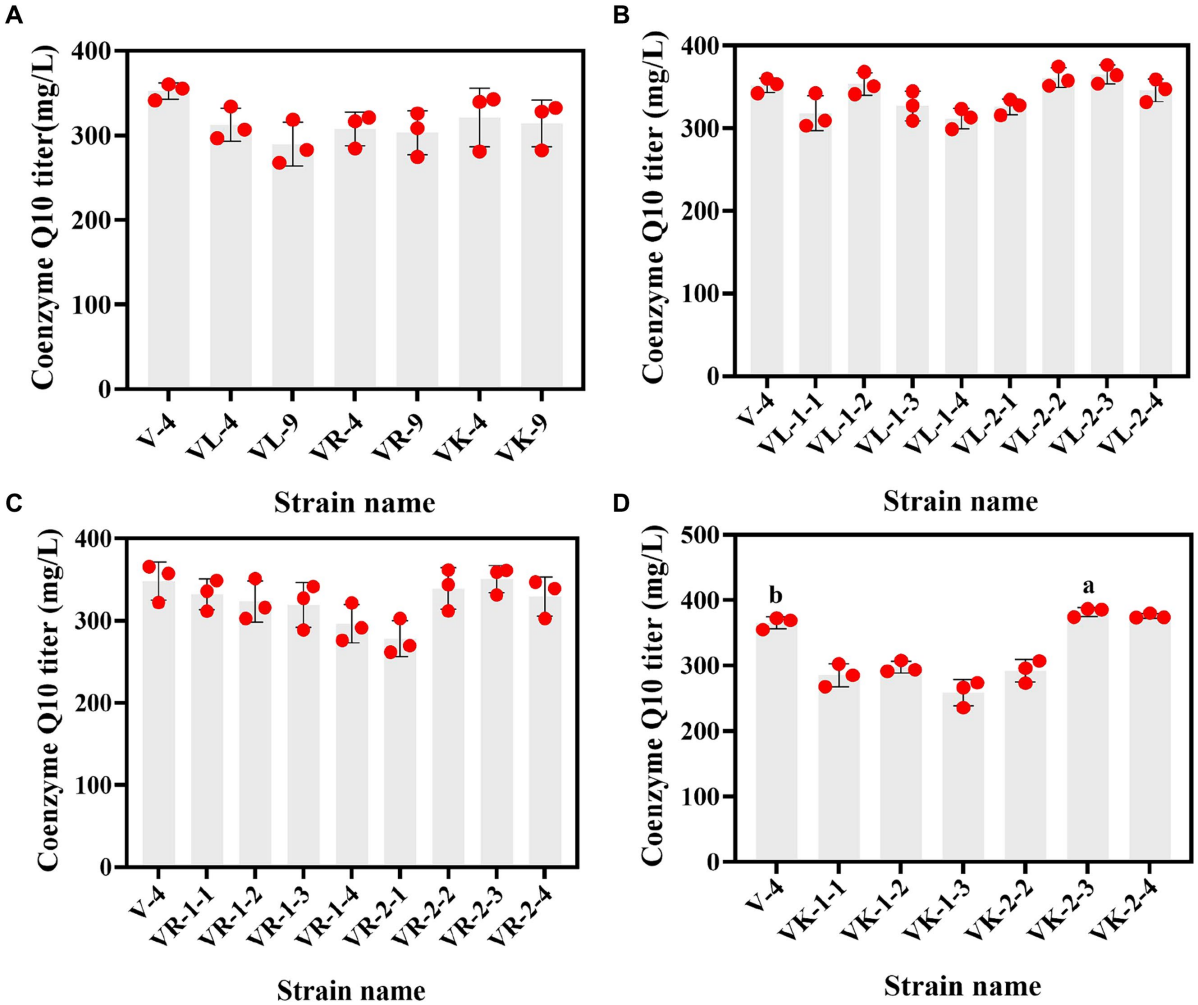
The relationship between dual-resistant mutant strains and the CoQ10 titer. **(A)** Dual-resistance mutant strains were obtained via ^12^C^6+^ heavy ion secondary mutagenesis. **(B–D)** are vitamin K3 and chloramphenicol, vitamin K3 and roxithromycin, and vitamin K3 and kanamycin dual-resistant mutant strains that were obtained under high-voltage prick electric field mutagenesis, respectively. The different lower-case letters in the shoulder note indicate that there was a significant difference between the two comparison combinations (p < 0.05, Student’s t-test).

The primary screening on these resistance plates was fast and suitable for the initial screening of mutant strains. Although the accuracy was not high, rescreening the initially screened strains by HPLC can balance efficiency and accuracy. However, due to the diverse genetic backgrounds of different *R. sphaeroides* strains, the factors limiting CoQ10 synthesis varied for each strain, leading to differing resistance plate results. In this study, V-0 underwent ^12^C^6+^ heavy ion primary mutagenesis, resulting in 52 resistant mutants on the resistant plate. By using HPLC for rescreening, we obtained 9 mutants with high CoQ10 titers. The CoQ10 titers of V-3, V-4, V-9, R-3, R-7, R-11, L-9, L-12, and K-1 were 355.07, 370.85, 358.68, 348.68, 330.63, 326.25, 355.64, 365.32, and 373.16 mg/L, respectively. Compared to those of the parent strain V-0 at the same fermentation period, the titers of CoQ10 increased to varying degrees, with the largest increase of 17.34% observed in the titer of V-4 CoQ10 (Figure 2). Afterward, V-4 underwent ^12^C^6+^ heavy ion secondary mutagenesis, resulting in the identification of 6 mutant strains with dual resistance through plate screening. However, compared to those of V-4, the titers of CoQ10 did not increase (Figure 3A), which may be due to V-4 developing tolerance, resulting in a poor mutagenic effect.

Subsequently, V-4 underwent high-voltage prick electric field mutagenesis and was combined with resistant substances for screening. This study yielded 22 resistant mutants on a dual-resistant plate, and one high-titer CoQ10 mutant was identified after HPLC rescreening. Named VK-2-3, the CoQ10 titer was 386.22 mg/L, representing a 4.15% increase compared to that of V-4 during the same fermentation period (Figures 3B–D). In addition, the CoQ10 titer of VK-2-3 was significantly higher than that of RS.VK and V-0. The CoQ10 titer of RS.VK was also significantly higher than that of V-0. These results suggest that the combination of heavy-ion beam and high-voltage prick electric field mutagenesis with resistance substances is more favorable for screening CoQ10 high-yielding strains (Supplementary Figure S3). In this study, complex mutagenesis of heavy ions and high-voltage prick electric fields combined with resistance screening significantly increased the CoQ10 titer of *R. sphaeroides* compared to traditional mutagenesis methods, addressing the issue of blunted mutagenesis effects (Ding et al., 2020; Li et al., 2022).

### 3.3 Cellular morphology of *Rhodobacter sphaeroides* by SEM

**Figure 4:**
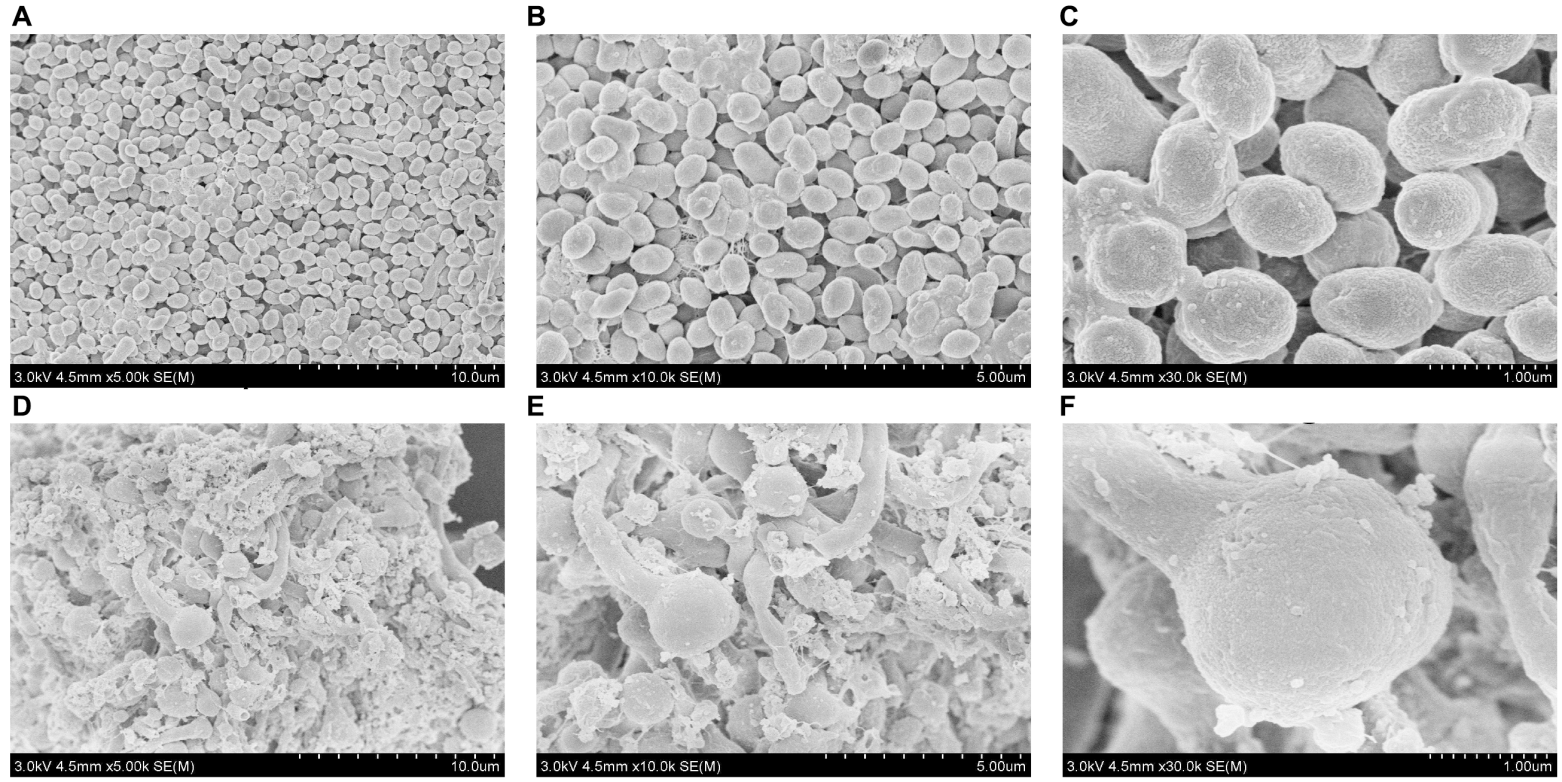
Scanning electron microscopic analysis of V-0 and VK-2-3 cells. **(A)** V-0 (Magnification = 5,000). **(B)** V-0 (magnification = 10,000). **(C)** V-0 (magnification = 30,000). **(D)** VK-2-3 (Magnification = 5,000). **(E)** VK-2-3 (Magnification = 10,000). **(F)** VK-2-3 (magnification = 30,000). The accelerating voltage was 3,000 V, and the working distance was 4,500 μm.

SEM primarily uses a small, focused electron beam to scan the surface of a sample. It is currently utilized in various life science fields, including pathology, developmental biology, and microanatomical structure research, and has attracted widespread attention (Müller et al., 2021). With the assistance of SEM, it is possible not only to observe the morphology of tissues or organs at a large scale and analyze the surface features of specific regions but also to reconstruct cells at the nanoscale. This has significantly advanced biomedicine and other related fields (Wang and Zhang, 2023). However, there are few reported studies on the use of SEM in *R. sphaeroides* mutant strains. In this study, scanning electron microscopy analysis revealed that the V-0 cells were closely arranged, had a regular morphology, and appeared in clusters. In contrast, VK-2-3 cells were tightly arranged but irregularly shaped, with a rougher surface and altered cell morphology (Figure 4). It was reported in the literature that when *silicate bacteria* were treated with ion beams, SEM analysis revealed that the morphology of the bacteria changed dramatically, which led to a significant increase in their ability to degrade potassium (Lu et al., 2006). Therefore, heavy ion beams and high voltage prick electric field mutations that roughened the cell surface and changed the morphology of VK-2-3 may be beneficial for the synthesis of CoQ10.

### 3.4 Identification of a segment of the differentially expressed NAD gene by comparative genomic analysis

With the in-depth study of cellular metabolic networks and the increasing maturity of metabolic engineering technology, the regulation of cellular metabolic pathways can significantly increase the yield of target products (Zhu et al., 2022). Furthermore, comparative genomics analysis can be utilized to analyze differences between genomes. Overexpressing or knocking out specific genes using genetic engineering techniques helps to clarify the molecular mechanisms underlying changes in target product yield (Li et al., 2013, 2021). To investigate the molecular mechanism behind the increased titer of VK-2-3 CoQ10, we used reverse metabolic engineering technology to study this issue. Mauve software was utilized to compare the sequencing results of the V-0 and VK-2-3 genomes. After analysis, the scaffold3 sequence in VK-2-3 was rearranged and classified into three sequences: scaffold5, scaffold31, and scaffold16 (Supplementary Table S2; Supplementary Figure S4). According to the annotation results, scaffold 31 was found to encode NAD, which is one of the three major enzyme systems in the synthesis pathway of CoQ10 and is a key enzyme (Kim et al., 2015). This led to the hypothesis that this might be one of the reasons for the increased production of CoQ10 in *R. sphaeroides*.

### 3.5 Effects of overexpressing and silencing the NAD gene on the CoQ10 titer

**Figure 5:**
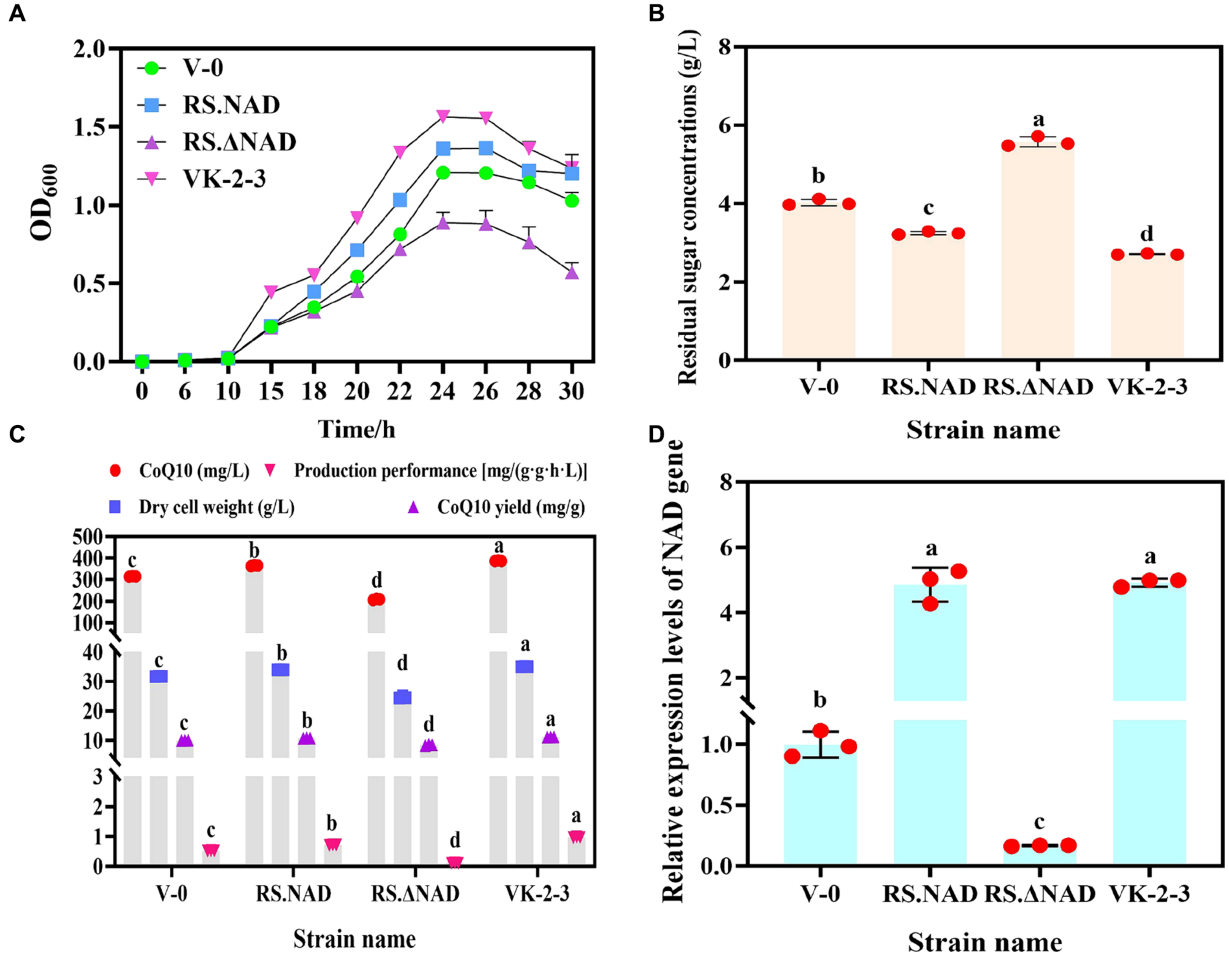
Determination of fermentation parameters and the relative expression of the NAD gene in V-0, RS.NAD, RS.ΔNAD, and VK-2-3 strains. **(A)** Growth curves of V-0, RS.NAD, RS.ΔNAD, and VK-2-3 strains. **(B)** The results of the V-0, RS.NAD, RS.ΔNAD, and VK-2-3 strains fermentation broth residual sugar tests. **(C)** Determination of the CoQ10 titer, yield, production performance, and dry cell weight of V-0, RS.NAD, RS.ΔNAD, and VK-2-3 strains. **(D)** Relative expression of the NAD gene in the fermentation broth of V-0, RS.NAD, RS.ΔNAD, and VK-2-3 strains. The different lower-case letters in the shoulder note indicate that there was a significant difference between the two comparison combinations (*p* < 0.05, Student’s *t*-test).

To determine whether rearranging the NAD gene is linked to an increase in the VK-2-3 CoQ10 titer, we overexpressed and silenced it in V-0, which is named RS.NAD and RS.ΔNAD, and fermentation experiments were conducted alongside V-0 and VK-2-3. The results indicated that V-0, RS.NAD, RS.ΔNAD, and VK-2-3 were in the lag phase at 0 ~ 10 h. The growth of VK-2-3 outperformed V-0, RS.NAD, and RS.ΔNAD, reaching the logarithmic phase after 10 h and significantly surpassing the growth of the other strains at approximately 24 h (Figure 5A). The overexpression of the NAD gene led to an increase in the number of bacteria per unit volume and time, similar to previous studies (Hou et al., 2007). After 72 h of fermentation, the residual sugar concentrations of V-0, RS.NAD, RS.ΔNAD, and VK-2-3 differed significantly. Compared to V-0, the residual sugar concentration decreased by 19.35 and 32.75% in RS.NAD and VK-2-3, and increased by 30.02% in the RS.ΔNAD fermentation broth (Figure 5B). The CoQ10 titer, dry cell weight, CoQ10 yield, and production performance of RS.NAD concentrations were 365.40 mg/L, 34.00 g/L, 9.93 mg/g, and 0.52 mg/(g·g·h·L); these values increased by 16.00, 7.15, 8.26, and 36.54%, respectively, compared with those of V-0. The CoQ10 titer, dry cell weight, CoQ10 yield, and production performance of RS.ΔNAD were 208.15 mg/L, 24.60 g/L, 8.46 mg/g, and 0.11 mg/(g·g·h·L). Compared with V-0, these values decreased by 33.92, 22.47, 14.80, and 78.85%, respectively. The CoQ10 titer, dry cell weight, CoQ10 yield, and production performance of VK-2-3 were 387.35 mg/L, 34.99 g/L, 11.07 mg/g, and 0.97 mg/(g·g·h·L); these values increased by 22.97, 10.27, 11.48, and 86.54%, respectively, compared with those of V-0 (Figure 5C). The RT–qPCR results indicated that the relative expression level of the NAD gene in the RS.NAD and VK-2-3 fermentation broth was 4.86 and 4.92 times greater than that in V-0, while the relative expression of the NAD gene in the RS.ΔNAD fermentation broth was 0.17 times greater than that in V-0 (Figure 5D). In this study, NAD-dependent dehydrogenase, a key enzyme in the synthesis of CoQ10 (Koo et al., 2010), was rearranged in VK-2-3 by compound mutagenesis. Both overexpression and silencing of this enzyme resulted in significant changes in the growth and glucose consumption of the strain; the level of its expression directly impacted the biosynthesis of CoQ10.

## 4 Conclusion

In summary, a VK-2-3 mutant strain with a high titer and yield of CoQ10 was obtained via combined mutagenesis with a ^12^C^6+^ heavy ion beam and a high-voltage prick electric field in this study. By applying reverse metabolic engineering techniques to analyze the mechanism of increased CoQ10 titer in the mutant strain VK-2-3, we found a rearrangement of the NAD gene in the genome of VK-2-3, which was experimentally confirmed to be the reason for the increased CoQ10 production in VK-2-3. However, the rearrangement of the NAD gene is only one of the reasons for the increased yield of CoQ10, and there are a number of potential genes that need to be further explored. This study provides a reference for analyzing the mechanism underlying the increase in the production of the target substance.

## Data availability statement

The datasets presented in this study can be found in online repositories. The names of the repository/repositories and accession number(s) can be found in the article/Supplementary material.

## Author contributions

LZ: Writing – review & editing, Writing – original draft, Validation, Formal analysis. L-yW: Writing – review & editing, Validation. Y-jH: Writing – review & editing, Resources. Y-xL: Writing – review & editing, Resources. Y-lL: Writing – review & editing, Software. J-hH: Writing – review & editing, Software. Z-jT: Writing – review & editing, Resources. Z-yL: Writing – review & editing, Supervision, Project administration, Funding acquisition, Conceptualization.
