# Supplementary material for: Analysis of the improved mechanism of Rhodobacter sphaeroides VK-2-3 coenzyme Q10 by reverse metabolic engineering
Source: Front Microbiol. 2024 Jul 4;15:1410505. doi: 10.3389/fmicb.2024.1410505 (PMC11254814; doi:10.3389/fmicb.2024.1410505)
Supplement: Supplementary file 1 [file Data_Sheet_1.docx]

Supplementary Material

Analysis of the improved mechanism of *Rhodobacter Sphaeroides* VK-2-3 coenzyme Q10 by reverse metabolic engineering

Long Zhang^1,2,3^, Le-yi Wang^3^, Yi-jun Han^4^, Yan-xin Liu^4^, Yong-li Li^1,2,3^, Jian-hua Hu^1,2,3^, Zhi-jie Tian^4^, Zhan-ying Liu^1,2,3^*

*** Correspondence: Zhan-ying Liu,** [**hgxylzy2008@imut.edu.cn**](mailto:hgxylzy2008@imut.edu.cn)

**1 Construction and transformation of recombinant plasmids**

Up-NAD (815 bp) and Down-NAD (933 bp) genes are homologous regions located upstream and downstream of the NAD gene (located between bases 147828 bp and 149042 bp of the V-0 genome), respectively. The NAD, Up-NAD, and Down-NAD gene fragments were obtained by PCR amplification using the V-0 genome as a template and purified with an agarose gel purification kit. Subsequently, the Up-NAD and Down-NAD genes were fused using overlapping PCR techniques. The plasmids and fragments used in this experiment were all digested with BamHI and HindIII for 4 h at 37°C for gel electrophoresis, and the products were recovered. The pBBR1MCS-4 and NAD genes were ligated overnight at 16°C using T4 DNA ligase and then transformed into *E. coli* competent cells. Subsequently, colony PCR identification and sequencing were performed. Overexpression plasmids were extracted from *E. coli* DH5α and transferred into the V-0 competence prepared in our laboratory using the electrotransformation method. Electrotransformation was carried out as follows:

1. The electrode cups were soaked in 75% alcohol for 15 min, washed with anhydrous ethanol for 10 min, rinsed twice with ddH_2_O, dried, and then placed in an ice bath.
2. A total of 1 µL of recombinant plasmid was added to 100 µL of V-0 competent cells without mixing. The mixture was added to the grooves of the two electrode pads in the electrode cup and then placed in an ice bath for 30 min.
3. The electrode cup was placed in the instrument, and the voltage was adjusted to 0.8, 1, 1.2, 1.4, and 1.8 kV. An electric shock was administered for 5.4-5.8 ms. The electrode cup was removed, and 800 μL of LB medium was added. The bacteria were then cultured with shaking at 32°C and 220 rpm for 2 h. A total of 50 μL of the sample was evenly spread onto medium containing Amp resistance and cultured upside down at 32°C for 5-6 d.

The V-0 competence was prepared as follows:

1. The V-0 bacterial solution was diluted and spread onto media A to cultivate a single colony. After selecting a single colony with robust growth, it was cultured in 100 mL of medium B overnight at 32°C with 220 rpm.
2. The bacterial solution was inoculated at 1% and transferred to 50 mL of media B. The culture was shaken at 32°C with 220 rpm until the OD600 reached 0.58-0.7, and then placed in an ice bath for 10 min. Separate the bacterial solution into two pre-cooled 50-ml centrifuge tubes.
3. Centrifuge at 4°C, 4500 r/min for 10 min, and discard the supernatant. Add 15 ml of pre-cooled 0.1 mol/L CaCl_2_-MgCl_2_ solution to each tube, gently pipette to resuspend the cells, and place in an ice bath for 10 min.
4. Centrifuge at 4°C, 4500 r/min for 10 min, and discard the supernatant. Add 1 ml of pre-cooled 0.1 mol/L CaCl_2_-MgCl_2_ solution to each tube, gently pipette to resuspend the cells, and place in an ice bath for 10 min.
5. Add 0.25 ml of pre-cooled sterile glycerin to each tube, ice bath for 10 min, and distribute into 20 1.5 mL sterile centrifuge tubes, dispense 100 μl per tube, and store in the refrigerator at -80°C for reserve.

**2 Methods of conjugation transfer of V-0**

1. V-0 and *E. coli* S17-1 (including knockout plasmid) were inoculated in media B and LB liquid media (LB liquid media with Kan resistance) and cultured to the logarithmic growth phase, respectively.
2. Each 1 ml of bacterial solution was placed in a 1.5 ml EP tube, centrifuged at 8000 r/min for 3 min, and the supernatant was then discarded.
3. The bacterial precipitate was washed once with 1 ml of 0.9% normal saline.
4. The bacteria were resuspended in the appropriate amount of fresh medium B (OD600 between 0.8 and 1.2).
5. The two types of bacterial solutions were mixed in varying proportions, with a total volume of 400 μL. The mixed bacterial solution was slowly absorbed, placed in the center of a sterile filter membrane on a plate, and then dried on the ultra-clean bench.
6. The lawn on the plate was washed with 500 µl of sterile water, spread on media A (Kan, 50 µg/mL; K_2_TeO_3_, 150 µg/mL), and then incubated in an inverted culture at 32℃ until black transformants appeared.
7. After inoculating the black transformants in media B (Kan, 50 µg/mL) and culturing them at 32°C and 220 rpm for 24 h, genomic DNA was extracted as a template. Primers CX-F/Down-NAD-R and Up-NAD-F/CX-R were used to verify the accuracy of the single-exchange strains.
8. A single clone of the verified correct single-exchange strain was selected and inoculated in media B (without resistance) and then incubated at 32°C and 220 rpm for 24 h.
9. The transfer was conducted using a 1% inoculation amount, double-exchange strains were screened, and the bacterial solution was diluted to a suitable concentration (10^–6^). Next, 50 μl of the diluted bacterial solution was spread on SMM solid media (containing 10% sucrose) and incubated at 32°C until single colonies appeared.
10. After picking single clones, they were inoculated in media B and cultured at 32°C and 220 rpm for 24 h. Genomic DNA was extracted as a template, and the primers CX-F and CX-R were used to verify the correctness of the double-exchange strain, which was then sequenced. The correct transformants were stored in glycerol at -80°C.

**SMM Media Formulation**: 20 mL of A solution, 1 mL of B solution, 4 g of DL malic acid, 3.5 g of K_2_HPO_4_, 2 g of KOH, 0.5 g of (NH_4_)_2_SO_4_, and 0.5 g of NaCl were fixed to 1 L with deionized water, adjusted to a pH of 6.9, and sterilized at 121 °C for 15 min, while 2% agar powder was added to the solid media.

**A solution:** 10 g of aminotriacetic acid and 8 g of KOH were added to 500 mL of deionized water. After completely dissolving, add 14.6 g of MgSO_4_, 1.7 g of CaCl_2_·2H_2_O, and 50 mL of C solution in that sequence. Finally, the pH was adjusted to 7.0.

**B solution:** 0.004 g biotin, 0.2 g nicotinic acid, and 0.1 g thiamine hydrochloride were dissolved in 100 mL of deionized water, fixed to 200 mL, and then filtered to remove bacteria.

**C solution:** 2 g EDTA, 11 g ZnSO_4_·7H_2_O, 5 g FeSO_4_·7H_2_O, 1.5 g MnSO_4_·H_2_O, 0.4 g CuSO_4_·5H_2_O, 0.12 g H_3_BO_3_ and 0.37 g CoCl_2_·6H_2_O were fixed to 1 L with deionized water.

**3 Screening of CoQ10 high-yielding mutants of *R. sphaeroides* by HPLC**

(1) Preparation of samples: Keeping the entire operation shielded from light, the fermentation broth, which had been allowed to reach room temperature, was thoroughly shaken and then precisely pipetted 5 mL into a 50 mL brown volumetric flask. First, add 1 mol/L hydrochloric acid (0.5 mL) for acidification. Then, add 0.5 mL of 30% hydrogen peroxide solution and an appropriate amount of anhydrous ethanol. Place the mixture in an ultrasonic cleaner for 2 to 3 min until all the bubbles are exhausted and the fermentation broth is free of condensation. The anhydrous ethanol was added to fix the volume. The water temperature was maintained at 55-60°C, and ultrasonic crushing was carried out for 45 min. After the ultrasound was completed and cooled to room temperature, it was then filtered using a 0.22 μm organic system filter membrane. The filtrate was collected in a brown bottle for HPLC detection.

(2) Testing conditions: CoQ10 was quantified using an HPLC system equipped with a C18 column (4.6 mm × 150 mm × 5 μm) and a UV detector at 275 nm. Methanol/ethanol (65:35, v/v) was used as the mobile phase at 35°C with a flow rate of 1.0 mL/min. Quantification was conducted by calculating the peak area using the standard external method.

Table S1. Primer sequences of the target gene

| Gene name | Primer sequences | Note |
| --- | --- | --- |
| *NAD-*F | 5′-CGGGATCCATGTCGAAGATCAAGGTAGC-3′ | For amplification of CDS sequence |
| *NAD-*R | 5′-ATAAGCTTTCAGGCGCCGAGCGC-3′ |  |
| Up-*NAD-*F | 5′-CGGGATCCCTCCGGTCGAAGAAG-3′ | For amplification of upstream and downstream homology arms of NAD gene |
| Up-*NAD-*R | 5′-TTCACTCTGGAAGGAGAGGCTCCC-3′ |  |
| Down*-NAD-*F | 5′-GGGAGCCTCTCCTTCCAGAGTGAA-3′ |  |
| Down*-NAD-*R | 5′-ATAAGCTTTGGTGGTATTTCCACCGCCAC-3′ |  |
| CX-F | 5′-ATCGCGAACTACTATCAGGGCTC-3′ | Primers for knockout validation |
| CX-R | 5′-TATGTGATCGTGACGCTCAAGGC-3′ |  |
| RT-*rpoZ-*F | 5′-TTCGAGCTGGTGATGCT-3′ | For relative expression analysis of *NAD* gene |
| RT-*rpoZ-*R | 5′-ACTCGATCTGGGTCTGG-3′ |  |
| RT-*NAD*-F | 5′-CTACGAGGAGGAATTCGCCG-3′ |  |
| RT-*NAD*-R | 5′-TCATCAGCACGCTCGTCATC-3′ |  |

Note: Underlining indicates the enzyme digestion site: GGATCC-BamHI, AAGCTT-HindIII

Table S2. List of site information for collinearity comparison

| Target ID | Target start | Target end | Query ID | Query start | Query end | Dir | Target size | Query size | Target length | Query length |
| --- | --- | --- | --- | --- | --- | --- | --- | --- | --- | --- |
| Scaffold1 | 1 | 1055993 | Scaffold1 | 1 | 1055902 | + | 0 | 0 | 1055993 | 1055902 |
| Scaffold10 | 28 | 7632 | Scaffold10 | 149817 | 157421 | + | 1 | 2 | 7605 | 7605 |
| Scaffold10 | 7633 | 157448 | Scaffold10 | 1 | 149816 | + | 2 | 1 | 149816 | 149816 |
| Scaffold11 | 1 | 145242 | Scaffold11 | 1 | 145242 | + | 3 | 3 | 145242 | 145242 |
| Scaffold12 | 1 | 143550 | Scaffold12 | 43 | 143592 | + | 4 | 4 | 143550 | 143550 |
| Scaffold13 | 1 | 94597 | Scaffold13 | 1404 | 96000 | + | 5 | 5 | 94597 | 94597 |
| Scaffold14 | 1 | 70751 | Scaffold14 | 1 | 70751 | + | 6 | 6 | 70751 | 70751 |
| Scaffold15 | 1 | 69675 | Scaffold15 | 1 | 69675 | + | 7 | 7 | 69675 | 69675 |
| Scaffold16 | 1 | 64680 | Scaffold17 | 1 | 64680 | + | 8 | 9 | 64680 | 64680 |
| Scaffold17 | 1 | 53967 | Scaffold18 | 1 | 53967 | + | 9 | 10 | 53967 | 53967 |
| Scaffold18 | 1 | 27683 | Scaffold19 | 1 | 27683 | + | 10 | 11 | 27683 | 27683 |
| Scaffold19 | 1 | 21295 | Scaffold20 | 1 | 21295 | + | 11 | 13 | 21295 | 21295 |
| Scaffold2 | 1 | 505790 | Scaffold2 | 1 | 505790 | + | 12 | 12 | 505790 | 505790 |
| Scaffold20 | 1 | 13962 | Scaffold22 | 1 | 13962 | + | 13 | 16 | 13962 | 13962 |
| Scaffold21 | 28 | 10911 | Scaffold21 | 5024 | 15907 | + | 14 | 15 | 10884 | 10884 |
| Scaffold22 | 1 | 7344 | Scaffold23 | 1 | 7344 | + | 15 | 17 | 7344 | 7344 |
| Scaffold23 | 1 | 5759 | Scaffold25 | 1 | 5759 | + | 16 | 20 | 5759 | 5759 |
| Scaffold24 | 1 | 5216 | Scaffold24 | 1 | 5216 | + | 17 | 18 | 5216 | 5216 |
| Scaffold25 | 1 | 5023 | Scaffold21 | 1 | 5023 | + | 18 | 14 | 5023 | 5023 |
| Scaffold26 | 1 | 4186 | Scaffold26 | 1 | 4186 | + | 19 | 21 | 4186 | 4186 |
| Scaffold27 | 1 | 3704 | Scaffold27 | 1 | 3704 | + | 20 | 22 | 3704 | 3704 |
| Scaffold28 | 1 | 3003 | Scaffold28 | 1 | 3003 | + | 21 | 23 | 3003 | 3003 |
| Scaffold29 | 1 | 2654 | Scaffold29 | 1 | 2654 | + | 22 | 24 | 2654 | 2654 |
| Scaffold3 | 1 | 308740 | Scaffold5 | 7 | 308746 | + | 23 | 32 | 308740 | 308740 |
| Scaffold3 | 308741 | 309338 | Scaffold31 | 240 | 837 | + | 24 | 27 | 598 | 598 |
| Scaffold3 | 309339 | 377819 | Scaffold16 | 1 | 68397 | + | 25 | 8 | 68481 | 68397 |
| Scaffold30 | 1 | 1956 | Scaffold30 | 1 | 1956 | + | 26 | 26 | 1956 | 1956 |
| Scaffold31 | 21 | 855 | Scaffold32 | 1 | 835 | + | 27 | 28 | 835 | 835 |
| Scaffold32 | 1 | 726 | Scaffold24 | 5217 | 5942 | - | 28 | 19 | 726 | 726 |
| Scaffold33 | 1 | 751 | Scaffold33 | 1 | 751 | + | 29 | 29 | 751 | 751 |
| Scaffold34 | 1 | 567 | Scaffold34 | 1 | 567 | + | 30 | 30 | 567 | 567 |
| Scaffold4 | 1 | 377024 | Scaffold3 | 1 | 377024 | + | 31 | 25 | 377024 | 377024 |
| Scaffold4 | 377025 | 377187 | Scaffold7 | 1 | 163 | - | 32 | 34 | 163 | 163 |
| Scaffold5 | 1 | 353859 | Scaffold4 | 1 | 353876 | + | 33 | 31 | 353859 | 353876 |
| Scaffold6 | 1 | 295375 | Scaffold6 | 1 | 295375 | + | 34 | 33 | 295375 | 295375 |
| Scaffold7 | 44 | 283523 | Scaffold7 | 164 | 283643 | + | 35 | 35 | 283480 | 283480 |
| Scaffold8 | 1 | 266112 | Scaffold8 | 1 | 266112 | + | 36 | 36 | 266112 | 266112 |
| Scaffold9 | 1 | 199750 | Scaffold9 | 21 | 199722 | + | 37 | 37 | 199750 | 199702 |

Note: Target ID: Target sequence name; Target start: the starting point of the target sequence alignment; Target end: the end of target sequence alignment; Query ID: Query sequence name; Query start: the starting point of query sequence alignment; Query end: the endpoint of query sequence alignment; Dir : comparison direction; Target size: the order of small comparison blocks on the target; Query size: query sequence alignment length; Target length: target sequence alignment length; Query length: query sequence alignment length.


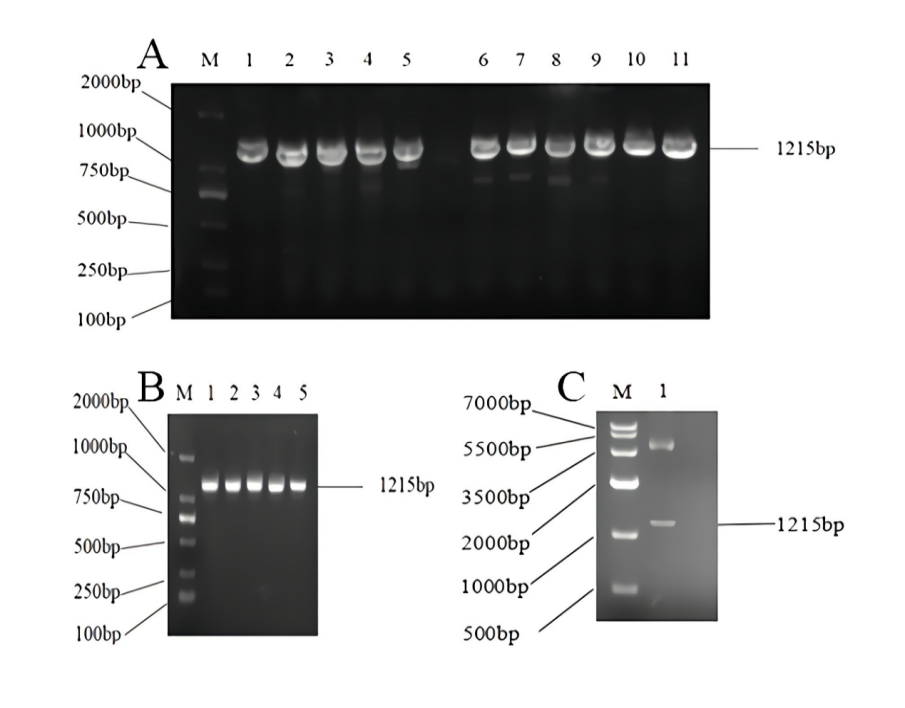


Figure S1. The results of the construction of the recombinant strain. (A) Results of nicotinamide adenine dinucleotide-dependent dehydrogenase (NAD) gene amplification. (B) NAD gene double digestion recovery results. (C) The results of the validation of double digestion of the pBBR1MCS-4-NAD plasmid.


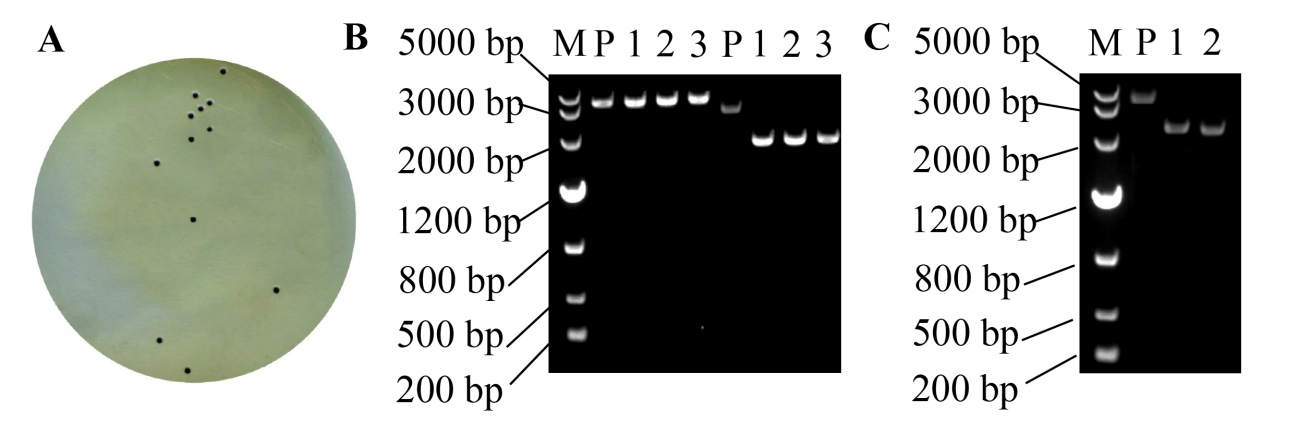


Figure S2**.** Screening and verifying NAD gene knockout strains. (A) Screening single-exchange transformants. (B) The single-exchange transformants were confirmed using the primers CX-F/Down-NAD-R (the first four gene bands) and Up-NAD-F/CX-R (the last four gene bands). (C) Double-exchange transformants were screened using CX-F/CX-R primers.

Note: P served as the positive control. In Figure S2-B, a single exchange in the upstream arm of the transformants is evident, with a 1,215 bases difference compared to the positive control. Figure S2-C shows that the strain completed the double exchange, differing by 1,215 bases from the positive control.


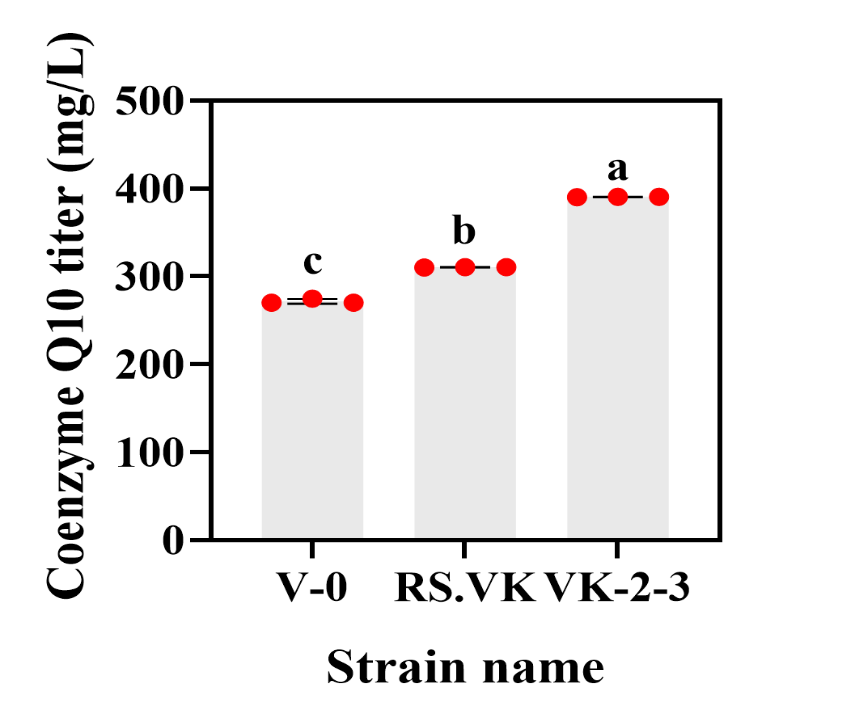


Figure S3. Coenzyme Q10 titers of V-0, RS.VK, and VK-2-3 strains. The different lower-case letters in the shoulder note indicate that there was a significant difference between the two comparison combinations (P<0.05, Student's t-test).


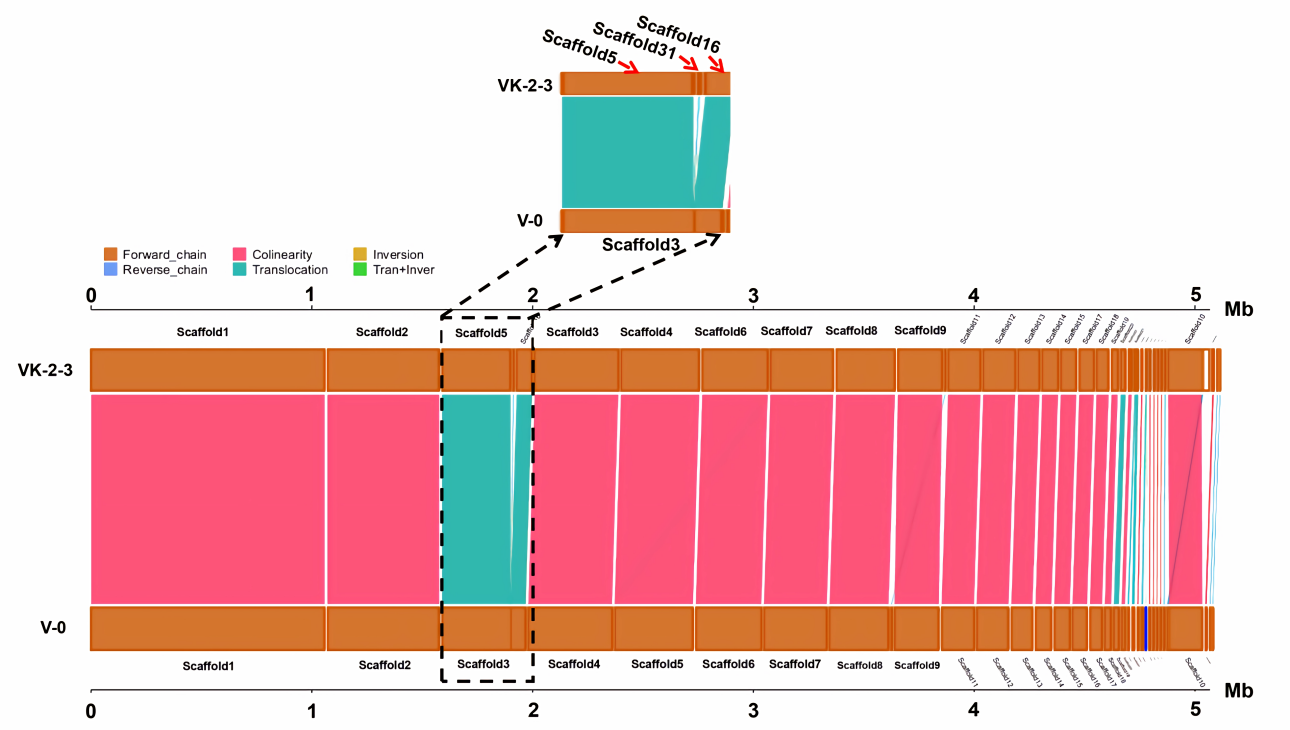


Figure S4. Diagram of collinearity analysis of the V-0 and VK-2-3 genomes.
